# Twenty-four-hour physical activity patterns associated with depressive symptoms: a cross-sectional study using big data-machine learning approach

**DOI:** 10.1186/s12889-024-18759-5

**Published:** 2024-05-07

**Authors:** Saida Salima Nawrin, Hitoshi Inada, Haruki Momma, Ryoichi Nagatomi

**Affiliations:** 1 Laboratory of Health and Sports Sciences, Tohoku University Graduate School of Biomedical Engineering, Sendai, Miyagi Japan; 2 Department of Medicine and Science in Sports and Exercise, Tohoku University Graduate School of Medicine, Sendai, Miyagi Japan; 3 Present Address: Department of Biochemistry & Cellular Biology, National Center of Neurology and Psychiatry, Kodaira, Tokyo, Japan

**Keywords:** Activity pattern, Depressive symptoms, Kernel K-means, Objectively measured physical activity, Time-series clustering, Unsupervised machine learning

## Abstract

**Background:**

Depression is a global burden with profound personal and economic consequences. Previous studies have reported that the amount of physical activity is associated with depression. However, the relationship between the temporal patterns of physical activity and depressive symptoms is poorly understood. In this exploratory study, we hypothesize that a particular temporal pattern of daily physical activity could be associated with depressive symptoms and might be a better marker than the total amount of physical activity.

**Methods:**

To address the hypothesis, we investigated the association between depressive symptoms and daily dominant activity behaviors based on 24-h temporal patterns of physical activity. We conducted a cross-sectional study on NHANES 2011–2012 data collected from the noninstitutionalized civilian resident population of the United States. The number of participants that had the whole set of physical activity data collected by the accelerometer is 6613. Among 6613 participants, 4242 participants had complete demography and Patient Health Questionnaire-9 (PHQ-9) questionnaire, a tool to quantify depressive symptoms. The association between activity-count behaviors and depressive symptoms was analyzed using multivariable logistic regression to adjust for confounding factors in sequential models.

**Results:**

We identified four physical activity-count behaviors based on five physical activity-counting patterns classified by unsupervised machine learning. Regarding PHQ-9 scores, we found that evening dominant behavior was positively associated with depressive symptoms compared to morning dominant behavior as the control group.

**Conclusions:**

Our results might contribute to monitoring and identifying individuals with latent depressive symptoms, emphasizing the importance of nuanced activity patterns and their probability of assessing depressive symptoms effectively.

**Supplementary Information:**

The online version contains supplementary material available at 10.1186/s12889-024-18759-5.

## Background

Depression is a mood disorder that causes persistent sadness and loss of interest in enjoyable activities [1–4]. Depression is a prevalent mental health condition affecting about 280 million people worldwide and responsible for more than 47 million disability-adjusted life years in 2019 [5, 6]. It is also a leading cause of disability globally and is associated with premature mortality from other illnesses [7] and suicide [8]. Depression also imposes substantial economic burdens. In 2010, it was estimated that approximately $US210.5 billion was spent annually in the US alone on lost work productivity and medical treatment associated with depression [9–11]. The manifestation of depressive symptoms is an important sign of the onset of depression [2, 4]. Therefore, understanding the prevalence of depressive symptoms within the population is vital for effective public health planning [12].

Physical activity is a factor associated with depression [13]. Recent observational studies found that every additional hour of light-intensity physical activity decreased the chances of being depressed [14, 15]. Similarly, higher physical activity levels, especially among women, are associated with a reduced likelihood of having depression [16]. Consistent with these observations, studies involving interventions have demonstrated that all modes of physical activity effectively reduce depressive symptoms, although high-intensity physical activity shows more significant improvements in reducing mild to moderate symptoms of depression, anxiety, and psychological distress in diverse adult populations [17].

While recent investigations challenge the notion that a higher volume or intensity of physical activity is beneficial for depressive symptoms [18, 19], some studies have indicated a potential association between the timing of physical activity and depressive symptoms [20, 21], possibly due to the shift in the circadian rhythm experienced by individuals with depression [22]. The shift in the circadian rhythm could result in changes in physical, mental, and behavioral patterns throughout the 24-h cycle, leading to a change in preferred times for engaging in activities among depressive populations [23, 24]. Therefore, the physical activity pattern in terms of timing could be a helpful and vital parameter in understanding the association between physical activity and depressive symptoms.

Several studies have explored the relationship between physical activity patterns, specifically in timing, and the association with depressive symptoms [20, 25–27]. By contrast, a previous study has reported that individuals with depression and anxiety exhibited lower overall activity levels but did not show a specific association between the timing of activity and the presence or severity of depressive symptoms [28]. No differences in the timing of physical activity between depressed and non-depressed individuals were found, even when participants receiving antidepressant treatment were excluded from the analysis [29]. Considering these reports, the association between physical activity patterns and depressive symptoms remains inconsistent.

The inconsistency of the association between depressive symptoms and physical activity patterns, including timing or phase, might be due to the difficulty of establishing a standardized procedure to categorize physical activity patterns. Previous studies can be attributed to the limitations in capturing the behavior of physical activity based on the temporal shape of the activity for each day [20, 26–29]. By aggregating data from multiple days, the unique temporal shape of each day's activity is diluted, potentially masking the associations with depressive symptoms [30]. These temporal shapes could be critical because depressive individuals have more fragmented physical activity throughout the day because of distorted circadian rhythm [30, 31]. Thus, the temporal shape of physical activity could be a valuable marker for detecting depressive symptoms [32].

Recently, machine learning (ML) has gained increasing prominence in the field of epidemiology due to its potential for effective disease prediction and patient care [32–35]. ML is also applied to detecting physical activities in daily behaviors such as sleeping, sitting, walking, ascending stairs, descending stairs, and running from big data obtained from wearable devices or smartphones [36]. Raw physical activity data is often complex and multidimensional. Therefore, ML analyses are beneficial even when relationships or interactions between variables are nonlinear [37–39]. In addition, another advantage of the ML approach is to enable us to measure accurate physical activity over a long period, possibly leading to figuring out physical activity patterns, which could be challenging to handle and unrecognized by manual assessments [32–35].

In this study, we investigated the relationship between activity behaviors, defined as the relative frequency of temporal physical activity patterns, and depressive symptoms using big data of the accelerometer from the National Health and Nutrition Examination Survey (NHANES) 2011–2012 [40] by time-series analysis with ML [41]. Here, we identified five 24-h activity-counting patterns and four physical activity-count behaviors. The 24-h activity-counting patterns were classified by temporal physical activity patterns for 24 h, reflecting a distinguishable activity pattern throughout the day. On the other hand, the physical activity-count behaviors were determined by the relative frequencies of the physical activity-counting patterns, and they would indicate a major physical activity pattern that each individual engages in throughout the week. Individuals with the Evening dominant behavior had a higher prevalence of depressive symptoms than those with the Morning dominant behavior (as a reference), even though they showed similar total activity count. These findings could highlight the importance of distribution in activity behaviors for understanding the relationship between physical activity and depressive symptoms. Recognizing the patterns and variability of physical activity might contribute to finding a behavioral context for manifesting depressive symptoms.

## Methods

In our cross-sectional study, we used data from NHANES 2011–2012, focusing on the noninstitutionalized civilian resident population of the United States. The study involved 6,917 participants who were given accelerometers to measure physical activity. Of these, 6,613 participants provided a complete 7-day data set of physical activity, resulting in 46,291 days (7 days for each participant: 7 × 6,613 = 46,291). We employed unsupervised machine learning by Kernel K-means to cluster the twenty-four-hour activity counting pattern, and later, based on the dominant activity counting pattern, we clustered the activity counting behavior by k-means clustering.

In this study, the physical activity-count behaviors were determined by the relative frequency of the physical activity-counting patterns, which are classified as temporal shapes based on physical activity-counting within 24 h. The physical activity-count behaviors would provide us with insights into the specific type of physical activity pattern that each individual engages through a week, while the physical activity-counting patterns reflect how an individual's activity is distributed throughout the day, based on rhythms or lifestyle. We classified four distinct activity-count behaviors based on the dominant activity pattern. Individuals with the Evening dominant behavior had a higher prevalence of depressive symptoms than those with the Morning dominant behavior (as a reference), even though they showed similar total activity count.

4,242 of 6,613 participants had complete demographic information and depressive symptoms data using the Patient Health Questionnaire-9 (PHQ-9), a tool used to assess depressive symptoms. Assessment using the PHQ-9 was performed once during data collection. Our subsequent analysis focused on these 4,242 participants. To explore the relationship between activity-count behavior and depressive symptoms, we conducted a multivariable logistic regression analysis on this subset of participants. We used cluster labels obtained from 6,613 participants, including those without a complete set of the demography and the PHQ-9, to generalize the dominant activity counting pattern and to minimize the selection bias depending on the response rate. The detailed participant flow and analysis overview are explained in a flow chart in Supplementary Figure S1.

### Activity counting data

National Health and Nutrition Examination Survey (NHANES) 2011–2012 data was used for the analysis [40]. The NHANES 2011–2012 received approval from the National Center for Health Statistics (NCHS) Ethics Review Board (Protocol #2011–2017). Participants were asked to wear a wrist-worn ActiGraph model GT3X + accelerometer for seven consecutive days, 24 h a day, starting on the day of their examination [42]. The wrist-worn ActiGraph model GT3X + accelerometer captured acceleration along three axes (x, y, and z) with 80 Hz sampling intervals as raw data in the original NHANES survey (PAX80_G Doc in [40]). The raw data was used to calculate the count data per minute of the physical activity monitor on the same website (Physical Activity Monitor—Minute in [40]). We used the count data per minute provided on the NHANS website as a starting data set to produce “counting data” per hour. Based on a previous study, the ActiGraph model GT3X + accelerometer has been reported as a reliable tool for capturing information on physical activity in free-living conditions over an extended duration. The study indicates that the accuracy of data obtained is higher when the accelerometer is worn continuously for 7 days, as opposed to a single-day wear scenario [43]. In this study, the device was worn on the non-dominant wrist, and participants were instructed to wear it continuously except when they needed to temporarily remove it. In our study, we exclusively included participants who provided complete data for the entire 7-day duration without resorting to any imputation techniques. This approach ensured that only individuals with a full set of data for each day were considered in our analysis.

### Depression data

The NHANES 2011–2012 uses the PHQ-9 questionnaire to measure depression. The Patient Health Questionnaire (PHQ-9) is a commonly used screening tool to assess the presence and severity of depression. It consists of nine questions about the frequency of symptoms of depression experienced over the past 2 weeks. Each question has four response categories: “not at all,” “several days,” “more than half the days,” and “nearly every day.” Each response category is assigned a point ranging from 0 to 3, with 0 indicating that the symptom was not present and 3 indicating that the symptom was present nearly every day. The scores for each item are then summed to give a total score ranging from 0 to 27. The final follow-up question in the PHQ-9 assesses the overall impairment caused by depressive symptoms. This question asks the patient to rate the degree to which their symptoms have caused problems in their daily life, including work, school, or relationships. Overall, the PHQ-9 provides a simple and reliable method for identifying individuals experiencing depression and assessing the severity of their symptoms. It is widely used in clinical practice and research to screen for depression and monitor the response to treatment. PHQ-9 score > or = 10 had a sensitivity of 88% and a specificity of 88% for major depression [44–46].

### Software and data processing

MATLAB (2021a, MathWorks, MA) and Python (3.11.0) were used for data processing and visualization.

### Twenty-four-hour activity counting pattern and daily activity counting behaviors

**Fig. 1:**
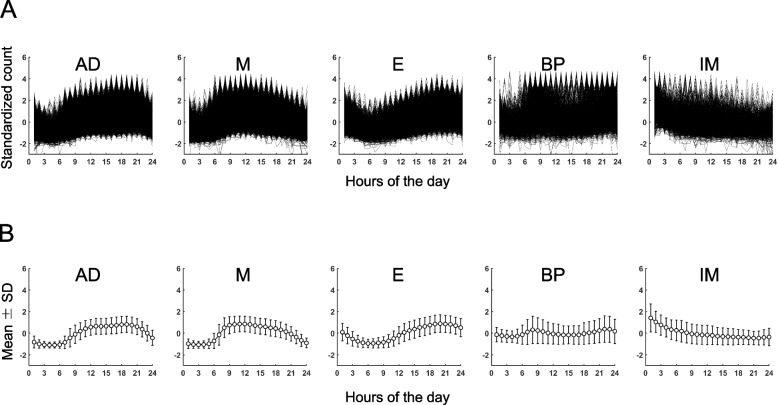
Five 24-h physical activity patterns. **A** Individual plots of standardized activity count for each pattern. **B** Plots of mean values with standard deviation (SD) for each pattern. AD, All-day; M, Morning; E, Evening; BP, Bi-phasic; IM, Irregular morning

**Fig. 2:**
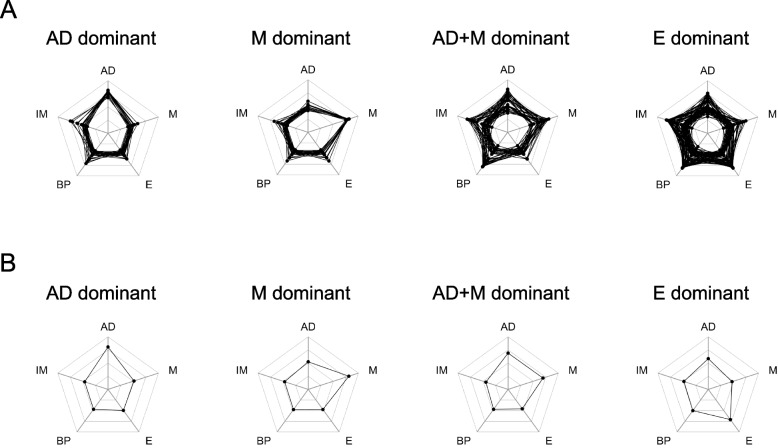
Four daily behavioral patterns. **A** Individual spider plots of the relative frequency of 24-h physical activity patterns. **B** Spider plots of mean values for each pattern. AD (All day) dominant; M (Morning) dominant; AD + M (All day + Morning) dominant; E (Evening) dominant

In this analysis, the minute-level physical activity data from the NHANES 2011–2012 dataset is utilized. Only participants with a whole day of data for 7 complete days (totaling 6613 participants) are included to ensure data completeness. The data is then converted into a standardized format (mean = 0 and SD = 1) for each 24 h, resulting in a dataset comprising 46,291 days of 24-h standardized data. To cluster the 24-h physical activity patterns, unsupervised ML such as Kernel K-means using the tslearn Python package is employed [47]. The physical activity-count behaviors were obtained as follows: 1) for each participant, the relative frequency of each 24-h physical activity-counting pattern (AD, M, E, BP, and IM) was calculated from the proportion of each pattern for 7 days. 2) To obtain the activity-count behaviors, vectors of the relative frequencies of each pattern (five variables for each participant) were clustered by unsupervised ML using a K-means clustering algorithm with MATLAB. The cluster names were determined according to the major patterns with higher relative frequency contained in each cluster. Figure 1 displays five clusters representing different 24-h physical activity patterns: all day (AD), morning (M), evening (E), bi-phasic (BP), and irregular morning (IM). Figure 2 illustrates four clusters of daily activity-count behaviors, categorized as AD dominant, M dominant, AD + M dominant, and E dominant.

### Depression measurement

The study used the PHQ-9 questionnaire to measure depression. Out of the total 6613 participants, 4,242 participants provided complete answers to all nine questions, and they have the complete dataset for Age, Gender, BMI, and Work situation. The questionnaire provides a total score ranging from 0 to 27, with a threshold score of 10 being used to determine depression [48]. Participants who scored less than 10 were labeled as not having depression, while those who scored 10 or more were labeled as having depression. Out of 4242 participants, 390 participants have depressive symptoms, and 3852 participants have no depressive symptoms.

### Statistical analysis

The statistical analyses were performed using JMP software (version 16.2.0). For the demographic analysis, we reported descriptive statistics such as means and standard deviations (SD) for continuous variables and percentages for categorical variables stratified by activity-count behavior. To compare differences in continuous variables, we used ANOVA, while chi-square tests were employed for categorical variables across different activity-count behaviors.

To investigate the relationship between activity-count behavior and depressive symptoms, we conducted a multivariable logistic regression analysis. Depressive symptoms were the dependent variable, and activity-count behavior was the independent variable. We calculated odds ratios (ORs) and 95% confidence intervals (CIs). Initially, we examined the crude model (Model 1), which assessed the association between activity-count behavior and depressive symptoms without considering any confounding factors. Subsequently, we introduced one confounding factor at a time, progressing from Model 2 to Model 6.

In order to assess the impact of residual confounding, we conducted a sensitivity analysis. This analysis aimed to determine the influence of age, BMI, gender, work situation, and total activity on the association between activity-count behavior and depressive symptoms. We performed logistic regression analyses similar to the previous models, adding only one individual confounding factor in each model. We used the Wald test to assess the significance of the association in both cases. We also checked the correlations between each individual variable. We considered statistical significance as *p* < 0.05 for all analyses.

## Results

### Demographic characteristics of activity count behavior

Twenty-four-hour activity-counting patterns and daily activity-count behaviors were determined using the ML procedure reported previously [41]. Five 24-h activity-counting patterns all day (AD), morning (M), evening (E), bi-phasic (BP), and irregular morning (IM) and 4 clusters of daily activity-count behaviors (AD dominant, M dominant, AD + M dominant, and E dominant) were identified (Figs. 1 and 2 and Supplementary Tables S1-S3). The daily activity-count behaviors were named based on the higher relative frequency of specific 24-h activity-counting patterns after clustering using unsupervised ML (Supplementary Table S3). For instance, AD dominant showed a 0.7900 ± 0.1281 of the relative frequency of AD pattern. M and E showed 0.8341 ± 0.1303 and 0.4916 ± 0.2396, respectively. AD + M dominant contains AD and M in similar proportions (0.4342 ± 0.1340 and 0.4396 ± 0.1168). Participants with a complete set of demography and PHQ-9 data (*n* = 4242) were extracted from the clustered data (*n* = 6613) and used in subsequent analyses. Table 1 presents the demographic characteristics of each activity-count behavior. Out of 4242 participants, M dominant behavior held the maximum number of participants (*n* = 1444, 34.04%), followed by AD + M dominant (*n* = 1075, 25.34%), AD dominant (*n* = 1012, 23.86%), and E dominant (*n* = 711, 16.76%) behaviors. For age, M dominant is the oldest adult (55.82 ± 16.43 years), followed by AD + M dominant (47.47 ± 17.60 years), AD dominant (44.68 ± 18.52 years), and E dominant (34.75 ± 15.76 years) (*p* < 0.0001). BMI does not show a significant difference among the activity-count behavior (*p* = 0.1284), and the BMI is highest for the AD + M dominant (29.18 ± 7.03) and lowest for the AD dominant (28.49 ± 7.09). E dominant showed the highest percentage of male participants (55.27%), followed by M dominant (52.22%) and AD + M dominant (49.12%). AD dominant has the lowest percentage of male participants (44.47%). The work situation also differs between activity-count behaviors (*p* < 0.0001). The total activity is highest in AD dominant (13114.39 ± 3606.71), followed by M dominant (12844.19 ± 4140.83), AD + M dominant (11960.01 ± 4292.37), and E dominant (11544.57 ± 4926.13). E dominant showed the highest prevalence of depressive symptoms (15.75%), followed by AD + M dominant (8.56%), AD dominant (8.40%), and M dominant (6.99%) (*p* < 0.0001).

**Table 1:** Demographic characteristics of the clusters

**Cluster**		**AD dominant**	**M dominant**	**AD+M dominant**	**E dominant**	**Total**	***P* value**
Number (%)		1012 (23.86)	1444 (34.04)	1075 (25.34)	711 (16.76)	4242	<0.0001
Age (Mean ± SD)		44.68 ± 18.52	55.82 ± 16.43	47.47 ± 17.60	34.75 ± 15.76	4242	<0.0001
BMI (Mean ± SD)		28.49 ± 7.09	29.01 ± 6.15	29.18 ± 7.03	28.83 ± 7.96	4242	0.1284
Gender	Male	44.47 %	52.22 %	49.12 %	55.27%	2125	<0.0001
	Female	55.53 %	47.78 %	50.88 %	44.73 %	2117	
Work situation	Working at a job or business	46.34 %	52.63 %	53.95 %	47.40 %	2146	<0.0001
	With a job or business but not at work	2.47 %	1.45 %	1.12 %	1.69 %	70	
	Looking for work	7.02 %	2.63 %	3.81 %	9.42%	217	
	Not working at a job or business	44.17 %	43.28 %	41.12 %	41.49 %	1809	
Total activity (m/s^2^) (Mean ± SD)		13114.39 ± 3606.71	12844.19 ± 4140.83	11960.01 ± 4292.37	11544.57 ± 4926.13		<0.0001
Depressive symptoms	Yes	8.40 %	6.99 %	8.56 %	15.75 %	390	<0.0001
	No	91.60 %	93.01 %	91.44 %	84.25 %	3852	

Descriptive statistics, including means (±SD) for continuous variables and percentages for categorical variables, are presented, stratified by activity-count behavior. Differences were assessed using ANOVA for continuous variables and chi-square tests for categorical variables. Significance was considered at *p* < 0.05

### Relationship between activity-count behavior and depressive symptoms

Table 2 represents the odds ratios (ORs) and 95% confidence intervals (CIs) for the depressive symptoms based on activity-count behaviors. The M dominant behavior was chosen as a reference since the cluster had the largest population with the lowest prevalence of depressive symptoms and the most representative activity-counting behavior in this population (Table 1 and Supplementary Table S2). In the crude model (Model 1), the OR for depressive symptoms was 2.48 (95% CI: 1.86–3.30) for the E dominant behavior, 1.24 (95% CI: 0.92–1.67) for the AD + M dominant behavior, and 1.21 (95% CI: 0.90–1.64) for the AD dominant behavior. A significant association between E dominant behavior and depressive symptoms was observed after adjusting for covariates, including age, BMI, gender, work situation, and total activity (Model 2 to 6). No strong correlation was observed among these covariates (Supplementary Figure S2). A significant difference was observed after adjusting for each confounding factor (age, BMI, gender, work situation, and total activity). E dominant behavior remains associated with a higher prevalence of depressive symptoms compared to M dominant behavior (*p* < 0.0001), as shown in Supplementary Table S4. These results suggest that the association between E dominant behavior and depressive symptoms is robust against these confounding factors.

**Table 2:** Association between activity count behaviors and the prevalence of depressive symptoms among 4242 participants

	Activity count Behaviors			
	M dominant	AD dominant	AD+M dominant	E dominant
Model 1	Reference	1.21 (0.90,1.64)	1.24 (0.92,1.67)	2.48 (1.86,3.30)
		*p* = 0.1958	*p* = 0.1451	*p* < 0.0001
Model 2	Reference	1.32 (0.96,1.79)	1.32 (0.98,1.78)	2.88 (2.11,3.96)
		*p* = 0.0794	*p* = 0.0691	*p* < 0.0001
Model 3	Reference	1.31 (0.96,1.78)	1.28 (0.95,1.73)	2.80 (2.04,3.83)
		*p* = 0.0850	*p* = 0.1009	*p* < 0.0001
Model 4	Reference	1.24 (0.91,1.69)	1.25 (0.93,1.69)	2.82 (2.05,3.87)
		*p* = 0.1647	*p* = 0.1358	*p* < 0.0001
Model 5	Reference	1.09 (0.79,1.50)	1.19 (0.88,1.62)	2.30 (1.66,3.20)
		*p* = 0.5750	*p* = 0.2463	*p* < 0.0001
Model 6	Reference	1.09 (0.79,1.50)	1.17 (0.86,1.59)	2.21 (1.58,3.09)
		*p* = 0.5783	*p* = 0.3080	*p* < 0.0001

This table summarizes logistic regression analyses exploring the association between activity-count behavior and depressive symptoms. Models progress from the crude analysis (Model 1) to introducing individual confounding factors (Models 2–6). Values are presented as odds ratios (95% confidence intervals)

Model 1: Activity-count behavior (crude model)

Model 2: Activity-count behavior, age

Model 3: Activity-count behavior, age, BMI

Model 4: Activity-count behavior, age, BMI, gender

Model 5: Activity-count behavior, age, BMI, gender, work situation

Model 6: Activity-count behavior, age, BMI, gender, work situation, total activity

## Discussion

In this study, we first characterized physical activity-counting patterns by examining the temporal shape of physical activity that reflects how an individual's activity is distributed throughout 24 h. Then, we defined physical activity-count behaviors by identifying the dominant physical activity-counting patterns, showing the repetitive features of the physical activity that individuals engage in throughout the week. This procedure was achieved using unsupervised ML techniques. The current study identified five physical activity-counting patterns and four physical activity-count behaviors. Based on the identified physical activity-count behaviors, we revealed that individuals exhibiting E dominant (Evening dominant) behavior had a higher prevalence of depressive symptoms compared to those with M dominant (Morning dominant) behavior, irrespective of total activity count. This association was confirmed after adjusting for potential confounding factors. We also found that individuals with a higher prevalence of depressive symptoms have higher day-to-day variability.

Recently, studies using ML to analyze a huge data set of physical movements appeared with potential applications in epidemiology, public health, and human behavioral science [32–35]. The ML is applied to identify or categorize behaviors such as sleeping, lying, sitting, standing, walking, ascending or stairs, running, and other routine activities like biking, household chores, and yoga [36]. However, few studies have directly extracted or categorized temporal physical activity patterns by ML but could not differentiate between the temporal physical activity pattern and the intensity [49] or the total amount of physical activity [50]. Our study focused on categorizing each 24-h activity-count into several clusters directly and identifying the behavioral tendencies of each individual based on the relative frequency of dominant activity patterns. Our approach based on data-driven analysis could be more straightforward, insightful, and beneficial in handling temporal patterns and dominant behaviors of human physical activity directly, although a relatively large complete data set is required.

Previous studies showed various results regarding the association between depressive symptoms and physical activity patterns, such as the timing of activity [26, 27], consistent with our result that a specific temporal behavioral pattern (E-dominant) is associated with depressive symptoms. In these reports, the higher depression severity was linked to increased activity late at night in the calculated 24-h activity pattern [26], and the depressed participants had significantly lower physical activity counts during the daytime compared to healthy controls [27]. In contrast, the other observational and case–control studies have indicated no association between depressive symptoms and activity timing [28, 29]. Different classifications of the activity patterns due to the distinct procedures might result in inconsistent conclusions since these observational and case–control studies did not observe or detect the evening or nighttime activity patterns, possibly leading to no association of physical activity patterns with depressive symptoms.

In our results, the E pattern showed the highest activity between 6 p.m. and 9 p.m., and the E dominant behavior showed a higher prevalence of depressive symptoms. The association between E dominant behavior and the depressive symptom might reflect a disruption of circadian rhythm, which could play a crucial role in regulating mood and sleep–wake cycles since disruptions in the circadian rhythms, such as delayed sleep–wake timing, have been associated with increased risk of depressive symptoms and mood disorders [51]. Alternatively, individuals with depression may reduce daytime activity and compensate for total physical activity by increasing their evening activity when they feel energetic [52, 53]. The latter possibility might explain our result in which E dominant (risk group) and M dominant (control group) behavior showed a similar total count activity.

Many previous studies reported that there is a connection between depressive symptoms and low physical activity levels [20, 27, 28]. These findings suggested that total physical activity would be significant for the association with depressive symptoms. For instance, individuals with depression had lower 24-h physical activity levels compared to controls without depression, and those with acute depression exhibited a marginal significance towards later timing of daily activity peaks, particularly in the evening [20]. Similarly, individuals with depression displayed significantly lower physical activity levels between 7 a.m. and 10 p.m. compared to healthy controls, considering both timing and activity levels [27]. Additionally, the presence and severity of depressive and anxiety disorders were associated with reduced overall daily activity levels, but no significant association was found with the timing of activity [28]. In our study, E-dominant behavior exhibited a total activity count similar to AD + M-dominant behavior (Table 1). However, only E-dominant behavior was found to be associated with depressive symptoms compared to M-dominant behavior (Table 2). On the other hand, the AD dominant behavior has a higher amount of activity count compared to the M dominant behavior (Table 1), but the AD dominant behavior is not associated with depressive symptoms (Table 2). These results suggest that the physical activity behavior based on the dominant physical activity pattern might be a more significant parameter than the total amount of physical activity to explain the association with depressive symptoms in a particular procedure.

We also examined the day-to-day variability of activity patterns within different activity behaviors (Supplementary Table S3) and their relationship with depressive symptoms. We found that AD dominant, M dominant, and AD + M dominant behaviors exhibit the least day-to-day variability, with nearly 80% of their dominant activity patterns corresponding to their respective behaviors. However, the E dominant behavior shows considerably more variability, as only 49% of E patterns align with E dominant behavior, while the rest are distributed among other patterns (Supplementary Table S3). In this analysis, we observed that individuals with higher day-to-day variability (E dominant behavior) had a higher prevalence of depressive symptoms compared to those with less day-to-day variability in their activity behavior (AD dominant, M dominant, and AD + M dominant behavior) (Table 1). These findings indicate that the variability and temporal shape of activity patterns may play a significant role in the relationship between physical activity and depressive symptoms. This underscores the importance of considering these factors when seeking to understand the connection between physical activity and depressive symptoms.

Recent research has yielded valuable insights into the multifaceted influences on depressive symptoms across different life stages and factors, such as age, BMI, gender, and employment. Age was associated with a gradual increase in depressive symptoms, especially among older individuals [54]. Furthermore, a study on the relationship between depressive symptoms and BMI anticipated that overweight or obese individuals would experience more severe depressive symptoms, regardless of their racial background [55]. There are gender differences in depressive disorder, where women are more likely to have a higher prevalence of depression than men [56]. Unemployment also could be associated with the onset of clinical major depression [57]. In our study, however, the male ratio was the highest (male: female = 55.27:44.73 in %) in the E dominant cluster among other clusters (Table 1). On the other hand, the unemployment was similar between AD dominant and E dominant (7.02% and 9.42%, respectively), but the OR for depressive symptoms was not significant in the AD dominant. In addition, the OR for depressive symptoms was significantly higher even after gender and work situation were adjusted in the E-dominant cluster (Table 2). Therefore, gender and unemployment could have a minor contribution to depressive symptoms in our study. Our study uncovered a higher prevalence of depressive symptoms among individuals engaged in E-dominant behavior compared to those with M-dominant behavior, even after adjusting for confounding factors above. Our finding provides insight into the contribution of the physical activity pattern to understanding depressive syndrome, emphasizing the importance of individualized assessment of physical activity behavior to understand depressive symptoms.

The extent to which our machine-learning procedure could be generalized remains in future studies. However, our approach in this study could be applicable to other diseases or disorders in which physical activity could be closely related to the diagnosis. Temporal physical activity patterns might also indicate a meaningful link to cardiovascular disease (CVD) and obesity [49, 50]. Clustering temporal physical activity patterns by machine learning could enable comprehensive description or analysis at a higher resolution.

A major limitation of the current study is that the causal relationship between activity-count behavior and depressive symptoms could not be established because of the cross-sectional design. In addition, self-reported questionnaires were used to assess depressive symptoms. Although PHQ-9 used in this study has high sensitivity and specificity [44–46], we did not precisely diagnose the depression. A major limitation of this study is that about one-third (2,371) of the 6,613 participants used for the clustering had to be excluded from the analysis of depressive symptoms due to lacking complete demographic and PHQ-9 questionnaire data, possibly leading to selection bias and less generalizability. Moreover, participants were conscious that their physical activity was being monitored, possibly leading to the Hawthorne effect by which their behavior may change due to awareness [58]. Ubiquitous physical activity monitoring using devices such as smartphones daily would potentially minimize this bias and improve the study's reliability.

However, our study would provide a possible impact of physical activity patterns rather than the total amount of physical activity on the relationship with depressive symptoms. Evaluating the physical activity/behavioral patterns, such as temporal changes or relative frequency, may lead to valuable insights into the association between physical activity and depressive symptoms. Our findings also have a potential benefit to developing software for wearable devices alerting or reminding a risk to prevent the progression of depressive symptoms. These findings might have implications for improving the management and prevention of depressive symptoms in clinical and community settings.

## Conclusions

This research illuminates the intricate link between the temporal patterns of physical activity and the prevalence of depressive symptoms. Utilizing unsupervised machine learning for the categorization of 24-h activity patterns, it uncovers distinct daily behavioral patterns related to physical activity. A pivotal discovery of this study is the positive correlation between physical activity patterns predominantly in the evening and depressive symptoms, in contrast to morning-centric activities. This underscores the necessity of considering not only the aggregate amount but also the temporal distribution of physical activity in the context of mental health.

The methodology of this study, which incorporates objective data from accelerometers and sophisticated clustering techniques, brings a detailed perspective to the exploration of physical activity's role in depression. The correlation identified between the evening-dominant pattern and depressive symptoms, irrespective of the total amount of activity, highlights the importance of considering the timing and pattern of physical activity. These findings pave the way for personalized interventions, such as custom-tailored activity recommendations or notifications from wearable devices, aiming to reduce the likelihood of depressive symptoms. In addition, the application of these findings in both clinical and community environments could significantly improve strategies for the prevention and management of depressive symptoms.

### Supplementary Information

Supplementary Material 1. 

Supplementary Material 2. 

## Data Availability

The dataset supporting the conclusions of this article is available in the NHANES repository. https://wwwn.cdc.gov/nchs/nhanes/continuousnhanes/default.aspx?BeginYear=2011

## Abbreviations

AD: All day
M: Morning
E: Evening
BP: Bi-phasic
IM: Irregular morning
AD + M: All day + Morning
NHANES: National Health and Nutrition Examination Survey
PHQ-9: Patient Health Questionnaire-9
BMI: Body Mass Index
MATLAB: Matrix Laboratory
ML: Machine learning
ANOVA: Analysis of Variance
SD: Standard Deviation
OR: Odd Ratio
CI: Confidence Interval
