# Supplementary material for: Twenty-four-hour physical activity patterns associated with depressive symptoms: a cross-sectional study using big data-machine learning approach
Source: BMC Public Health. 2024 May 7;24:1254. doi: 10.1186/s12889-024-18759-5 (PMC11075341; doi:10.1186/s12889-024-18759-5)
Supplement: Supplementary file 1 — Supplementary Material 1. [file 12889_2024_18759_MOESM1_ESM.docx]

**Supplementary Figure S1. Flow chart.**

Participant Selection Flow Chart and Analysis Overview in NHANES 2011-2012


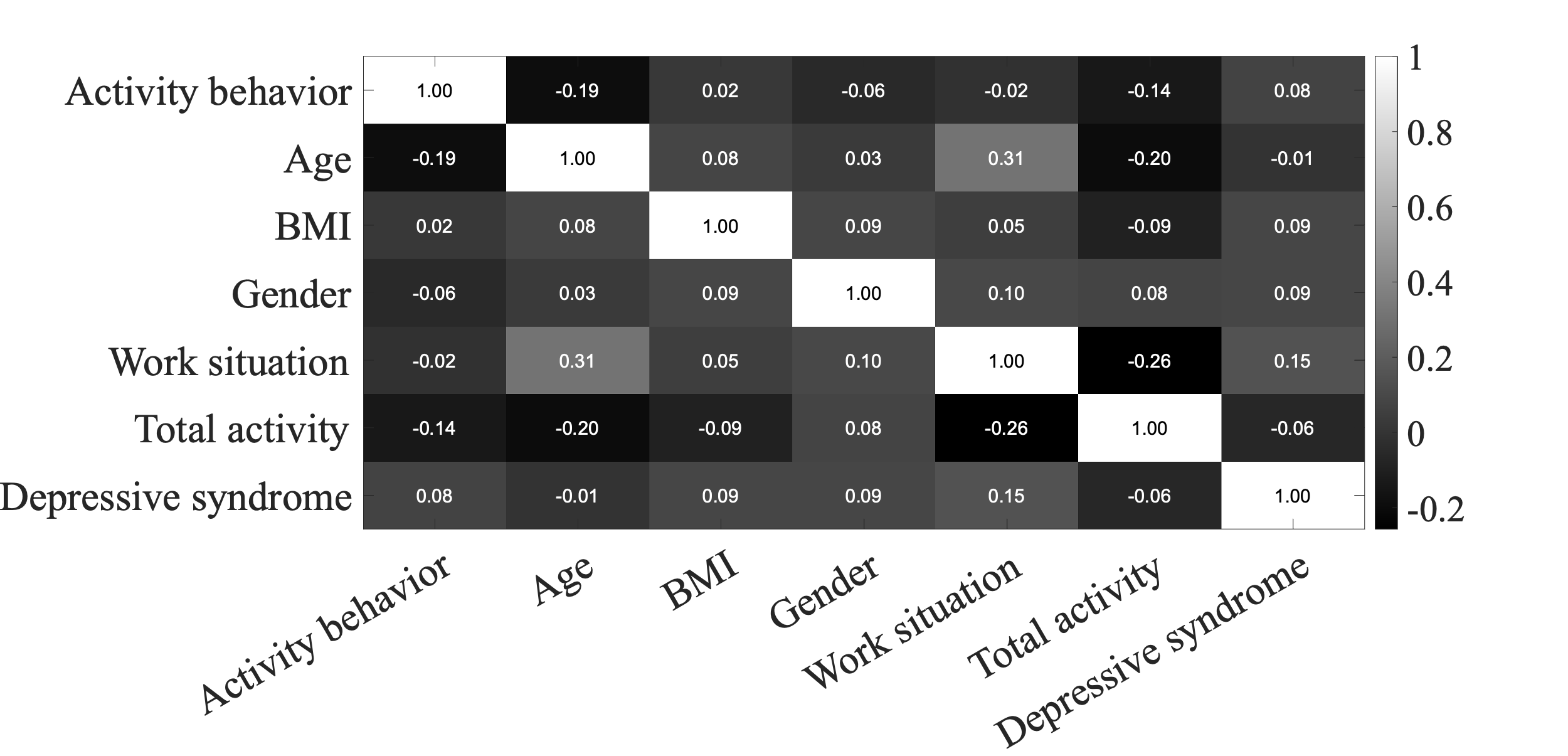


**Supplementary Figure S2. Correlation coefficients.**

Work situation and age showed a very weak positive correlation. The work situation and total activity showed a very weak negative correlation. Other variables showed no correlation among them.
