# Supplementary material for: Twenty-four-hour physical activity patterns associated with depressive symptoms: a cross-sectional study using big data-machine learning approach
Source: BMC Public Health. 2024 May 7;24:1254. doi: 10.1186/s12889-024-18759-5 (PMC11075341; doi:10.1186/s12889-024-18759-5)
Supplement: Supplementary file 2 — Supplementary Material 2. [file 12889_2024_18759_MOESM2_ESM.docx]

| **Table S1.** **Number of days for each activity-counting pattern (6613 participants x 7 Days)** | | | |
| --- | --- | --- | --- |
| Activity patterns | No of days | % of days | Daily activity count  (mean ± SD) |
| AD | 18980 | 41.00 | 14786.06 ± 5454.00 |
| M | 19156 | 41.38 | 14004.09 ± 5405.45 |
| E | 5033 | 10.87 | 12586.48 ± 5551.76 |
| BP | 1932 | 4.17 | 4640.34 ± 5755.53 |
| IM | 1190 | 2.57 | 7016.28 ± 7419.17 |
| Total | 46291 | 100 |  |

| **Table S2. Number of participants for behavior (6613 participants)** | | | |
| --- | --- | --- | --- |
| Activity-count Behavior | No of participants | % of participants | Total activity count  (mean ± SD) |
| AD dominant | 1792 | 27.09 | 14996.28 ± 4636.84 |
| M dominant | 1964 | 29.69 | 13628.09 ± 4594.38 |
| AD+M dominant | 1855 | 28.05 | 13307.74 ± 5063.63 |
| E dominant | 1002 | 15.15 | 11589.80 ± 5254.22 |
| Total | 6613 | 100 |  |

| **Table S3. Proportion of activity-counting patterns in each behavior** | | | | | |
| --- | --- | --- | --- | --- | --- |
| Activity-counting pattern relative frequency (Mean ± SD) | | | | | |
| Activity-count Behavior | AD | M | E | BP | IM |
| AD dominant | 0.7900 ± 0.1281 | 0.1201 ± 0.0192 | 0.0668 ± 0.1016 | 0.0140 ± 0.0499 | 0.0091 ± 0.0395 |
| M dominant | 0.1269 ± 0.1114 | 0.8341 ± 0.1303 | 0.0135 ± 0.0505 | 0.0175 ± 0.0580 | 0.0081 ± 0.0371 |
| AD+M dominant | 0.4342 ± 0.1340 | 0.4396 ± 0.1168 | 0.0433 ± 0.0782 | 0.0594 ± 0.1264 | 0.0236 ± 0.0707 |
| E dominant | 0.2407 ± 0.1772 | 0.0677 ± 0.0958 | 0.4916 ± 0.2396 | 0.1061 ± 0.1793 | 0.0940 ± 0.1604 |

| Table S4: Sensitivity analysis (Odds ratios for depressive symptoms by activity behavior for 4242 participants) | | | | |
| --- | --- | --- | --- | --- |
|  | Activity count behaviors | | | |
|  | M dominant | AD dominant | AD+M dominant | E dominant |
| Model 1 | Reference | 1.21 (0.90,1.64)  p = 0.1958 | 1.24 (0.92,1.67)  p = 0.1451 | 2.48 (1.86,3.30)  p < 0.0001 |
| Model 2 | Reference | 1.32 (0.96,1.79)  p= 0.0794 | 1.32 (0.98,1.78)  p= 0.0691 | 2.88 (2.11,3.96)  p < 0.0001 |
| Model 3 | Reference | 1.23 (0.91,1.66)  p= 0.1711 | 1.22 (0.91,1.64)  p= 0.1724 | 2.48 (1.87,3.31)  p < 0.0001 |
| Model 4 | Reference | 1.16 (0.86,1.57)  p= 0.3288 | 1.22 (0.90,1.64)  p= 0.1836 | 2.56 (1.92,3.41)  p < 0.0001 |
| Model 5 | Reference | 1.18 (0.87,1.60)  p= 0.2728 | 1.27 (0.95,1.72)  p= 0.1073 | 2.52 (1.89,3.38)  p < 0.0001 |
| Model 6 | Reference | 1.23 (0.92,1.67)  p= 0.1657 | 1.19 (0.89,1.61)  p= 0.2301 | 2.35 (1.76,3.14)  p < 0.0001 |
| Notes:  Model 1: Activity behavior (crude model)  Model 2: Activity behavior, age  Model 3: Activity behavior, BMI  Model 4: Activity behavior, gender  Model 5: Activity behavior, work situation  Model 6: Activity behavior, total activity | | | | |
